# Duplication of AKT2 Gene in Ovarian Cancer: A Potentially Novel Mechanism for Tumor-Induced Hypoglycemia

**DOI:** 10.7759/cureus.25813

**Published:** 2022-06-10

**Authors:** Hussam R Alkaissi, Zachary Mostel, Samy I McFarlane

**Affiliations:** 1 Internal Medicine, Kings County Hospital Center, Brooklyn, USA; 2 Internal Medicine, Veterans Affairs Medical Center, Brooklyn, USA; 3 Internal Medicine, State University of New York Downstate Medical Center, Brooklyn, USA; 4 Internal Medicine, Downstate-Health Science University, Brooklyn, USA

**Keywords:** ovarian tumor, akt2 duplication, insulin signaling, akt pathway, paraneoplastic syndrome, non-islet cell tumor hypoglycemia, tumor-induced hypoglycemia

## Abstract

Severe hypoglycemia occurs with different types of tumors, including islet cell and non-islet cell tumors. Non-islet cell tumor hypoglycemia (NICTH) is a rare and potentially life-threatening complication of malignancy. The primary underlying mechanism of NICTH proposed in the literature includes paraneoplastic overproduction of insulin-like growth factor-2 (IGF-2), the production of autoantibodies against insulin or its receptors, or the presence of extensive metastatic burden replacing hepatic tissue or adrenal glands. In this report, we propose a potentially novel mechanism underlying NICTH involving stimulation of the insulin signaling pathway in a 58-year-old woman with a rare ovarian tumor of Müllerian origin that carries a duplication of the AKT2 gene. AKT2 is a molecular mediator of insulin signaling. To our knowledge, this is the first reported case of tumor-induced hypoglycemia associated with AKT2 gene duplication. In this report also, we discuss the currently available diagnostic modalities and highlight the therapeutic rationale in patients with NICTH, a highly vulnerable population.

## Introduction

Tumor-induced hypoglycemia (TIH) is a notorious paraneoplastic syndrome seen in several malignant and benign tumors. TIH is most commonly seen in patients with insulinomas, but other non-islet cell tumors also induce hypoglycemia (NICTH) by several mechanisms. The most common type of NICTH is the overproduction of incompletely processed insulin-like growth factor-2 (IGF-2) that stimulates the insulin receptors leading to increased glucose uptake [1]. TIH may also result from autoantibodies against insulin or its receptors or an extensive tumor burden replacing hepatic tissues or adrenal glands where hemorrhage or tumor burden results in adrenocortical insufficiency with subsequent hypoglycemia.

Treatment varies according to the cause, from tumor resection to ameliorating symptoms with glucocorticoids and diazoxide [1]. Here we present a case of a female patient with an ovarian tumor of Müllerian origin who had recurrent severe hypoglycemic episodes and discuss the role of the genetic markers of the tumor that could potentially explain the underlying mechanism of hypoglycemia and its implication in the diagnosis and management of this life-threatening complications.

## Case presentation

A 58-year-old woman was brought in by emergency medical services (EMS) for altered mental status and hypoglycemia. The patient's daughter could not wake her mother for an outpatient appointment scheduled for that morning and called EMS. The patient was hemodynamically stable but unresponsive to verbal stimuli. Point-of-care (POC) capillary glucose was 22 milligrams/deciliter (mg/dL), and she was given 25 grams (g) of dextrose 50% by EMS; repeat POC glucose was 102 mg/dL with a return to her baseline mental status. She was brought to the emergency department for further evaluation. The patient had a history of worsening abdominal distention over the past few months and, as a result, had early satiety and mild shortness of breath. She did not use any hypoglycemic agents or insulin. She had no fevers, chills, abdominal pain, nausea or vomiting, changes in bowel movements, dysuria, or known sick contacts.

Her past medical history included clear-cell adenocarcinoma of the ovary with peritoneal carcinomatosis, hypertension, iron deficiency anemia, and peripheral neuropathy, possibly chemotherapy-related adverse reaction. Clear cell adenocarcinoma of the ovary was diagnosed six months prior to presentation. Exploratory laparotomy revealed bilateral adnexal masses, extensive peritoneal disease encasing the bowel loops with lesions on the urinary bladder, mesenteric shortening, and omental caking to the level of the liver. The decision was made to defer debulking of the tumor, given diffuse peritoneal and diaphragmatic involvement and the need for multiple resections of the bowel. Biopsy of the tumor specimen showed clear cell adenocarcinoma of Mullerian origin (International Federation of Gynecology and Obstetrics (FIGO) 2013 stage IIIC).

Immunocytochemistry of the tumor cells was positive for Paired Box 8 (PAX8), Cytokeratin 7 (CK7), caudal-type homeobox transcription factor 2 (CDX2, focal), and napsin (focal); the cells were negative for calretinin, estrogen receptor, and podoplanin (D2-40). Genomic studies of the ovarian tumor included the following mutations: tumor protein 53 (TP53) alteration in splice site 993+2T>A, F-Box and WD Repeat Domain Containing 7 (FBXW7) alteration, cyclin E1 (CCNE1) amplification, AKR thymoma gene 2 (AKT2) duplication, and mitogen-activated protein kinase kinase kinase 1 (MAP3K1) alteration. No breast cancer gene (BRCA) 1 or BRCA 2 mutations were identified. The patient was treated with four cycles of paclitaxel and carboplatin and three cycles of doxorubicin and bevacizumab. Of note, she was hospitalized two months prior with spontaneous bacterial peritonitis. Cytology of the peritoneal fluid showed atypical glandular cells consistent with adenocarcinoma of the ovary (positive for PAX8; negative for Wilms' tumor 1 gene [WT1] and p53).

Her past surgical history included appendectomy and biopsy of her ovary at the time of diagnosis. Her family history was non-contributory, with no ovarian, breast, or other cancer histories. Her home medications included amlodipine and gabapentin.

Vital signs were as follows: temperature 99 degrees Fahrenheit, blood pressure 130/75 mmHg, heart rate 103 beats per minute, oxygen saturation 99% on room air. Initial POC glucose was 79 mg/dL. The patient was cachectic with a large, distended abdomen on physical examination. The cardiopulmonary exam was without abnormality. The abdomen was soft, non-tender, distended, and positive for bowel sounds, fluid waves, and shifting dullness. There was 2+ pitting lower extremity edema to the bilateral knees and palpable dorsalis pedis pulses. She was able to tolerate oral feeding and repeat juice intake. Repeat POC glucose measurement was 30 mg/dL after six hours and 31 mg/dL after nine hours, and the patient was started on an intravenous (IV) infusion of dextrose 5%.

Laboratory findings were significant for hemoglobin 7.9 g/dL (near the patient's baseline), and a white blood cell count slightly up trending to 10,500 cell/mL without evidence of an infection, attributed to increased burden of malignancy. The comprehensive metabolic panel, thyroid function, and coagulation studies were within normal limits (Table 1).

**Table 1:** Laboratory data including comprehensive metabolic panel and complete blood count. eGFR: estimated glomerular filtration rate.

Variable	First admission	Second admission (1 month later)	Reference range
Sodium (mmol/L)	138	140	135-145
Potassium (mmol/L)	4.7	4	3.5-5.1
Chloride (mmol/L)	101	104	98-107
Carbon dioxide (mmol/L)	25	26	21-31
Urea nitrogen (mg/dL)	21	23	7-25
Creatinine (mg/dL)	0.69	0.7	0.6-1.2
eGFR (ml/min/1.73 m2)	101	99	>60
Total protein (g/dL)	5.4	5.1	6.4-8.3
Albumin (g/dL)	2.7	2.2	2.8-5.7
Aspartate aminotransferase (U/L)	21	35	10-35
Alanine aminotransferase (U/L)	<5	6	0-31
Total bilirubin (mg/dL)	0.2	0.2	0-1.2
Alkaline phosphatase (U/L)	83	91	25-125
White-cell count (per µl)	10,500	9,000	3,500-10,800
Hemoglobin (g/dl)	7.9	7.5	12-16
Platelets count (per µl)	453,000	390,000	130,000-400,000

**Figure 1:**
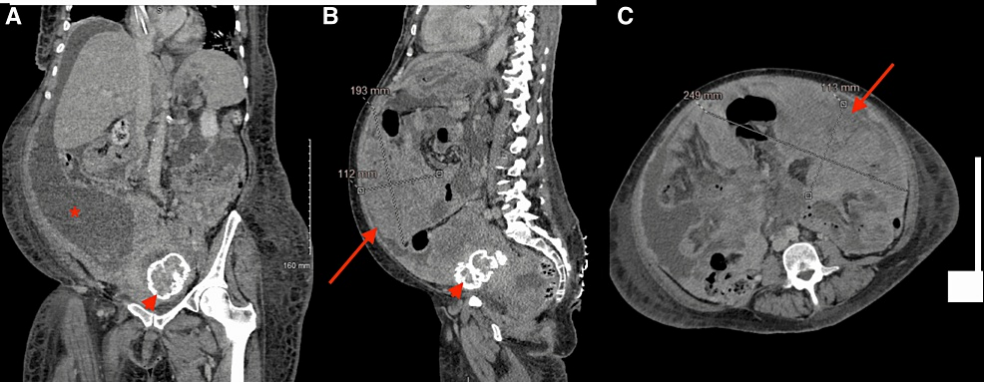
CT scan of the abdomen and pelvis. (A) A coronal section showing ascites (asterisk), the liver is seen without metastatic disease, normal size, and contour. (B) A sagittal section shows a large peritoneal mass (arrow) of 19 x 25 x 11 cm, also seen on (C) the axial section. Arrowhead points toward an incidental calcified fibroid seen in (A) and (B).

Human immunodeficiency virus (HIV), hepatitis B surface antigen and core antibodies, and hepatitis C antibody were all negative. ECG showed sinus tachycardia. CT of the abdomen and pelvis with contrast showed a large cystic right ovarian mass with extensive soft tissue nodularity; peritoneal carcinomatosis with extensive soft tissue nodules and masses extending along nearly all the peritoneal reflections, and ligaments with large volume ascites which had significantly progressed from the prior study; and no evidence of bowel obstruction (Figures 1A-1C). A transthoracic echocardiogram showed normal left ventricular wall motion and an ejection fraction of 60%.

Laboratory workup for hypoglycemia was obtained during the patient's admission showing random POC glucose 37 mg/dL, glucose on basic metabolic panel (BMP) was as low as 22 mg/dL, appropriately suppressed insulin and C-peptide levels, normal IGF-2 and IGBP-3, and normal morning cortisol (Table 2). Serum was negative for insulin antibodies and sulfonylureas.

**Table 2:** Laboratory data including hypoglycemia work up. IGF2: insulin-like growth factor 2, IGFBP3: IGF binding protein 3.

Variable	First admission	Reference range
Glucose (mg/dL)	37	80-140
Hemoglobin A1c	5.3%	4-5.6%
Insulin (mU/L)	0.2	2.6-24.9
Anti insulin antibodies	Negative	Negative
C-peptide (ng/mL)	0.1	1.1-4.4
Sulfonylurea	Undetectable	Undetectable
ß-hydroxybutyrate	0.07	<0.4
IGF2 (ng/mL)	711	267-616
IGFBP3 (µg/L)	1,930	2,238-5,717
Morning cortisol (µg/dL)	14.97	6.2-19.4
Thyroid-stimulating hormone (µIU/mL)	2.4	0.27-4.2

The patient continued to have persistent, recurrent hypoglycemia with POC glucose measurements as low as 30 mg/dL occurring multiple times per day despite good oral intake. Hypoglycemia was confirmed by low glucose levels on BMP as well, ranging between 30 and 55 mg/dL during the hypoglycemic episodes. Diazoxide 70 mg every eight hours was also started. The IV fluids were switched to dextrose 10%-0.9% normal saline. Diazoxide was titrated up to 180 mg every eight hours; oral Prednisone 5 mg every eight hours was added, then switched to hydrocortisone 50 mg IV every six hours. Pharmacologic management had a minimal impact on episodic hypoglycemia, diazoxide was titrated to 260 mg every eight hours, and prednisone was titrated to 60 mg daily. The patient's recurrent hypoglycemia persisted. However, she opted for discharge to home hospice, with her current medication regimen, and with continued POC glucose monitoring and she expired 48 hours later.

## Discussion

**Figure 2:**
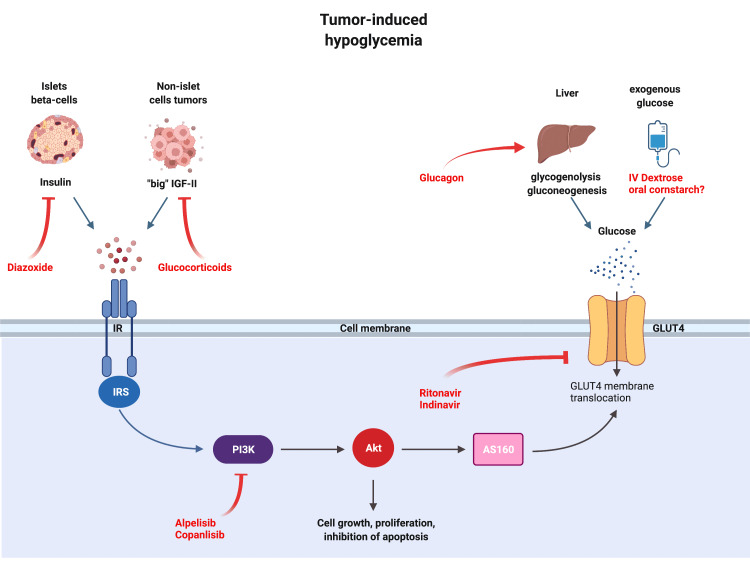
Mechanisms of tumor-induced hypoglycemia and treatment options. Some tumors activate the insulin signaling pathway (left half of the figure) by secretion various hormones such as insulin, GLP-1, and “big” IGF-II. Another way to cause hypoglycemia is by metastasizing to the liver, adrenals, and pituitary gland, organs that control glucose homeostasis (right half of the figure). AKT is seen in the middle, transmitting signals from the insulin receptor to translocate GLUT4 and internalize glucose. Treatment options are shown in red, such as glucocorticoids, diazoxide, glucagon, and dextrose infusion. Other possible options are also shown, such as protease inhibitors and PI3K inhibitors. AKT: AKR thymoma gene, AS160: AKT substrate of 160 kDa, GLUT4: glucose transporter 4, IR: insulin receptor, IRS: insulin receptor substrate, PI3K: phosphoinositide 3-kinase.

We present a patient with an unresectable ovarian clear-cell adenocarcinoma of Müllerian origin who developed recurrent severe hypoglycemic episodes, with capillary glucose virtually undetectable with levels as low as <10 mg/dL that resolved temporarily with the administration of glucose. These tumors are rare, affecting about 6 per million, and they arise from Müllerian structures ( including ovarian epithelial, fallopian, or peritoneal tumors). To our knowledge, they have not been reported to be associated with TIH before. The patient had suppressed insulin levels and normal IGF and IGFBP levels, with no evidence of hepatic or adrenal metastasis. She did not respond to high doses of glucocorticoids and diazoxide. The tumor carried duplication of the AKT2 gene, a key role player in mediating insulin signaling and cellular growth (Figure 2).

TIH can be broadly grouped under two main categories, hormone-mediated and non-hormone-mediated. Tumors secrete a myriad of hormones that can cause hypoglycemia as a part of paraneoplastic syndrome. These include insulin, insulin-like growth factor (IGF), somatostatin, and glucagon-like peptide 1 (GLP-1) [1].

Insulin-mediated hypoglycemia includes causes such as insulinoma, nesidioblastosis (beta-cell hyperplasia), and on rare occasions, extrapancreatic insulin-secreting tumors [1,2]. Very rarely, tumors secreting GLP-1 (GLP-1oma) can induce hypoglycemia through a GLP-1-mediated increase in insulin release [3]. Such patients have elevated insulin, proinsulin, and C-peptide, and they respond to treatment with diazoxide, a vasodilator with the additional property of inhibiting beta cells' insulin release [1]. Our patient had suppressed insulin, proinsulin, and C-peptide with no response to diazoxide treatment, ruling out insulin and GLP-1 mediated etiologies.

IGF2-mediated hypoglycemia is another group of TIH that is mediated through insulin signaling. Often referred to as NICTH, which is mediated by the release of an unprocessed IGF2 (pro-IGF2 or “big” IGF2) that suppresses growth hormone, IGF1, and IGFBP3 through negative feedback, and is thought to mimic insulin action in high concentrations [4,5]. It is diagnosed either by demonstrating high levels of IGF2 or an elevated IGF2/IGF1 ratio (ranging from 16 to 64) [6]. Patients respond to treatment of the underlying tumors or high doses of glucocorticoids [1]. Our patient had normal IGF2 and IGFBP3 levels; moreover, she did not respond to the high dose of steroids, thus making IGF-mediated hypoglycemia unlikely as an etiology.

Before moving away from the insulin signaling pathway, it is worth mentioning that in some patients with secretory B-cell malignancies, an exceedingly rare entity, that is the production of anti-insulin or anti-insulin receptor autoantibodies have been reported to stimulate the insulin receptor causing hypoglycemia [7,8]. Our patient had negative anti-insulin antibodies in her serum.

One of the counter-regulatory hormones to insulin action is glucagon, and in rare instances, reduced glucagon action can lead to hypoglycemia. In a single case report of ovarian neuroendocrine tumor, namely a somatostatinoma, hypoglycemia was part of a syndrome of new-onset diabetes, diarrhea, and steatorrhea, all mediated by somatostatin. The hypoglycemia was a product of the inhibitory effect of somatostatin on glucagon, thus losing the counter-regulatory effect against insulin [9,10]. Our patient had adenocarcinoma, with no evidence of neuroendocrine tissue on histopathology, with the absence of diarrhea and diabetes (other manifestations of somatostatin excess), making this an unlikely explanation for her hypoglycemia.

Non-hormonal causes of hypoglycemia include metastases to organs involved in glucose homeostasis, such as the liver, adrenals, and pituitary gland. In patients with extensive liver metastases, the endogenous glucose output (mediated by glycogenolysis and gluconeogenesis) is impaired [11,12]. Our patient had no liver metastases. Furthermore, she had normal cortisol and TSH levels, and treatment with glucocorticoids did not ameliorate the hypoglycemia.

Other non-hormonal causes can include tumor uptake of glucose. That was the initial hypothesis on why certain tumors cause hypoglycemia before discovering “big-IGF” [1,4]. We examined the tumor's molecular signature to find that it had a duplication of AKT2. AKT is a serine/threonine kinase that lies downstream from the insulin receptor (Figure 1) and transmits the signal to AS160, leading to the translocation of GLUT4 to the plasma membrane to facilitate cellular glucose uptake [13]. Such tumors, in theory, can continuously express GLUT4 and avidly consume glucose from the serum. Add to that, the heavy tumor burden (bilateral adnexal masses with extensive peritoneal metastatic disease) may contribute to the uptake of glucose.

A tumor uptaking glucose had been the prevailing theory of TIH until Daughaday reported IGF2-mediated hypoglycemia [4]. Glucose uptake is an inherent characteristic of many tumors that serves as the basis of positron emission tomography (PET scan) [1]. Ideally, a PET scan could have proven our theory by demonstrating avid glucose uptake into the tumors, but unfortunately, the patient died before undergoing a PET scan.

PI3K/AKT/mTOR axis is one of the most commonly involved molecular pathways in ovarian oncogenesis. AKT2 gene duplication is reported in 13%-18% of cases of primary ovarian cancers [14]. Its role is to increase glucose uptake, inhibit apoptosis, DNA repair and progress of the cell cycle [14,15]. AKT role in metabolism is crucial, as it conveys insulin (and other growth factors) signals to internalize glucose by expressing glucose transporters GLUT4 on cell membranes [13]. Hussain et al. reported three children with recurrent hypoglycemia caused by a germline gain-of-function of AKT2. Cellular studies showed constitutive recruitment of GLUT4 to the plasma membrane independent of insulin action in cells from those patients [16]. Similarly, Arya et al. reported another AKT2 gain of function with recurrent hypoglycemias and growth anomalies [17]. On the other hand, a loss of function of AKT2 in the mice model leads to severe insulin resistance, reduced glucose uptakes by myocytes, and hyperglycemia [18].

In most cases, glucocorticoids can ameliorate TIH when more radical treatment options for the underlying tumors are not feasible [1,19]. Unfortunately, our patient did not respond and was dependent on continuous IV dextrose infusion. This led us to consider other possible options that we would like to present here in dealing with recalcitrant TIH. Yet, one must be cautious as the evidence for these hypotheses is anecdotal at best or largely derived from basic research.

Nocturnal and fasting hypoglycemia is a constant threat to patients with glycogen storage disease type 1 (von Gierke). It is due to an enzymatic defect in glucose-6-phosphatase, any glucose produced from glycogenolysis or gluconeogenesis cannot be released from the cell; as such, recurrent hypoglycemia is a central feature of the disease [20]. One option used in patients with von Gierke disease is uncooked cornstarch given before sleep, which has been well studied in preventing hypoglycemia since 1984 [21]. Recently, cornstarch has been modified to extend fasting to 10 hours without hypoglycemia [22]. The inevitable recurrence of hypoglycemia in von Gierke disease and TIH are similar, though mechanistically and pathogenically different. Cornstarch was used by Aluri et al. in an 80-year-old female with an unresectable fibrous tumor who had recurrent hypoglycemic episodes. Similarly, euglycemia was difficult to maintain even with continuous glucose infusion. Therefore, she was started on uncooked cornstarch and prednisone, which prevented further hypoglycemic episodes [23].

Other options to prevent hypoglycemia may arise from understanding insulin signaling and how to interfere with it. For example, PI3K, a kinase that plays a central role in insulin signaling and growth, has been targeted by several inhibitors, such as alpelisib and copanlisib, FDA-approved for HR-positive, HER2-negative metastatic breast cancer [13,24]. These agents cause hyperglycemia, with rates as high as 63%, given their role in interfering with the insulin signaling pathway, and might be potentially beneficial in resistant cases of TIH [24].

Another class of medications known to induce hyperglycemia is protease inhibitors (PI) used in HIV treatment. PI can directly competitively inhibit GLUT4, and even a single dose of indinavir can induce a transient reversible state of insulin resistance [25,26]. A large study of HIV patients on PI showed that 14% developed diabetes, and up to 46% developed insulin resistance [27].

## Conclusions

In this report, we presented a case of TIH in a 58-year-old woman with ovarian cancer and AKT2 gene duplication. We discussed the underlying mechanisms of hypoglycemia in NICTH relating that to the therapeutic rationale of various treatment options. Treatment options vary according to the etiology, from resection of the tumor to palliative prevention of hypoglycemia, commonly with glucose infusion, glucocorticoids, and diazoxide. We also present anecdotal and other evidence supporting the use of other agents that could be potentially useful in the prevention of recurrent TIH.

## References

[1] Iglesias P, Díez JJ (2014). Management of endocrine disease: a clinical update on tumor-induced hypoglycemia. Eur J Endocrinol.

[2] Service FJ, McMahon MM, O'Brien PC (1991). Functioning insulinoma - incidence, recurrence, and long-term survival of patients: a 60-year study. Mayo Clinic Proc.

[3] Roberts RE, Zhao M, Whitelaw BC (2012). GLP-1 and glucagon secretion from a pancreatic neuroendocrine tumor causing diabetes and hyperinsulinemic hypoglycemia. J Clin Endocrinol Metab.

[4] Daughaday WH, Emanuele MA, Brooks MH, Barbato AL, Kapadia M, Rotwein P (1988). Synthesis and secretion of insulin-like growth factor II by a leiomyosarcoma with associated hypoglycemia. N Engl J Med.

[5] Dynkevich Y, Rother KI, Whitford I (2013). Tumors, IGF-2 and hypoglycemia: insights from the clinic, the laboratory and the historical archive. Endocrine Rev.

[6] Fukuda I, Hizuka N, Ishikawa Y (2006). Clinical features of insulin-like growth factor-II producing non-islet-cell tumor hypoglycemia. Growth Horm IGF Res.

[7] Redmon B, Pyzdrowski KL, Elson MK, Kay NE, Dalmasso AP, Nuttall FQ (1992). Hypoglycemia due to a monoclonal insulin-binding antibody in multiple myeloma. N Engl J Med.

[8] Braund WJ, Naylor BA, Williamson DH, Buley ID, Clark A, Chapel HM, Turner RC (1987). Autoimmunity to insulin receptor and hypoglycaemia in patient with Hodgkin's disease. Lancet.

[9] Todd JF, Stanley SA, Roufosse CA, Bishop AE, Khoo B, Bloom SR, Meeran K (2003). A tumour that secretes glucagon-like peptide-1 and somatostatin in a patient with reactive hypoglycaemia and diabetes. Lancet.

[10] Sugiyama T, Nakanishi M, Hoshimoto K (2012). Severely fluctuating blood glucose levels associated with a somatostatin-producing ovarian neuroendocrine tumor. J Clin Endocrinol Metabol.

[11] Yoshida D, Sugisaki Y, Tamaki T, Saitoh N, Node Y, Shimura T, Teramoto A (2000). Intracranial malignant meningioma with abdominal metastases associated with hypoglycemic shock: a case report. J Neurooncol.

[12] Vieweg WV, Reitz RE, Weinstein RL (1973). Addison's disease secondary to metastatic carcinoma: an example of adrenocortical and adrenomedullary insufficiency. Cancer.

[13] Saltiel AR (2021). Insulin signaling in health and disease. J Clin Invest.

[14] Ediriweera MK, Tennekoon KH, Samarakoon SR (2019). Role of the PI3K/AKT/mTOR signaling pathway in ovarian cancer: biological and therapeutic significance. Semin Cancer Biol.

[15] Nicholson KM, Anderson NG (2002). The protein kinase B/Akt signalling pathway in human malignancy. Cell Signal.

[16] Hussain K, Challis B, Rocha N (2011). An activating mutation of AKT2 and human hypoglycemia. Science.

[17] Arya VB, Flanagan SE, Schober E, Rami-Merhar B, Ellard S, Hussain K (2014). Activating AKT2 mutation: hypoinsulinemic hypoketotic hypoglycemia. J Clin Endocrinol Metab.

[18] Garofalo RS, Orena SJ, Rafidi K (2003). Severe diabetes, age-dependent loss of adipose tissue, and mild growth deficiency in mice lacking Akt2/PKB beta. J Clin Invest.

[19] Teale JD, Marks V (1998). Glucocorticoid therapy suppresses abnormal secretion of big IGF-II by non-islet cell tumours inducing hypoglycaemia (NICTH). Clin Endocrinol (Oxf).

[20] Froissart R, Piraud M, Boudjemline AM (2011). Glucose-6-phosphatase deficiency. Orphanet J Rare Dis.

[21] Chen YT, Cornblath M, Sidbury JB (1984). Cornstarch therapy in type I glycogen-storage disease. N Engl J Med.

[22] Correia CE, Bhattacharya K, Lee PJ (2008). Use of modified cornstarch therapy to extend fasting in glycogen storage disease types Ia and Ib. Am J Clin Nutr.

[23] Aluri VM, Julius BR, Langstengel JO (2017). Glucocorticoids and cornstarch therapy for non-islet cell tumor hypoglycemia: a case report. AACE Clin Case Rep.

[24] Cheung YM, McDonnell M, Hamnvik OR (2022). A targeted approach to phosphoinositide-3-kinase/Akt/mammalian target of rapamycin-induced hyperglycemia. Curr Probl Cancer.

[25] Vyas AK, Koster JC, Tzekov A, Hruz PW (2010). Effects of the HIV protease inhibitor ritonavir on GLUT4 knock-out mice. J Biol Chem.

[26] Noor MA, Seneviratne T, Aweeka FT (2002). Indinavir acutely inhibits insulin-stimulated glucose disposal in humans: a randomized, placebo-controlled study. AIDS.

[27] Lien LF, Feinglos MN (2005). Protease inhibitor-induced diabetic complications : incidence, management and prevention. Drug Saf.

